# The association between single-point insulin sensitivity estimator and high-sensitivity C-reactive protein with collateral circulation in coronary chronic total occlusion

**DOI:** 10.3389/fendo.2026.1835713

**Published:** 2026-05-22

**Authors:** Yazhao Sun, Xiao Yu, Chunlan Bai, Jie Huang

**Affiliations:** 1 Department of Cardiology, Cangzhou People’s Hospital, Cangzhou, Hebei, China; 2 Department of Neurology Intervention, Cangzhou People’s Hospital, Cangzhou, Hebei, China

**Keywords:** chronic total occlusion, collateral circulation, hs-CRP, insulin resistance, SPISE

## Abstract

**Background:**

The single-point insulin sensitivity estimator (SPISE) is a novel metric used to assess insulin resistance. However, its association with collateral circulation impairment in patients with chronic total occlusion (CTO) remains unclear. This study aims to investigate the relationship between SPISE, high-sensitivity C-reactive protein (hs-CRP), and collateral circulation impairment.

**Methods:**

Logistic regression models, restricted cubic spline (RCS) models, and subgroup analyses were utilized to explore the associations between SPISE, hs-CRP, and collateral circulation impairment. The predictive capability was evaluated using receiver operating characteristic (ROC) curves. Both base and extended models were constructed, and the net reclassification improvement (NRI) and integrated discrimination improvement (IDI) indices were calculated to assess the incremental predictive value of the additional biomarkers. A mediation effect model was employed to analyze the mediating role of hs-CRP in the relationship between SPISE and collateral circulation impairment. Additionally, the proportional scaling method was used to determine the weights of SPISE and hs-CRP, and a composite score was developed for sensitivity analysis.

**Results:**

A total of 362 participants were included in this study. After adjusting for all covariates, SPISE was negatively associated with collateral circulation impairment (OR = 0.371, 95% CI 0.245-0.543, P<0.001), while hs-CRP was positively associated with collateral circulation impairment (OR = 2.133, 95% CI 1.617-2.872, P<0.001). These associations remained consistent across most subgroups, with both SPISE and hs-CRP showing a linear relationship with collateral circulation impairment. The area under the curve (AUC) for SPISE in predicting collateral circulation impairment was 0.730 (95% CI 0.677-0.782), and the AUC for hs-CRP was 0.739 (95% CI 0.685-0.795). After adding SPISE and hs-CRP to the base model, the predictive ability of the extended model significantly improved, with the AUC increasing from 0.763 (95% CI 0.713-0.814) to 0.872 (95% CI 0.835-0.908). The continuous NRI was 0.794 (95% CI 0.592-1.096) and the IDI was 0.197 (95% CI 0.127-0.284). Mediation analysis indicated that hs-CRP partially mediated the relationship between SPISE and collateral circulation impairment, with a mediation proportion of 27% (P<0.001) after multivariable adjustment. Sensitivity analysis incorporating the SPISE-hs-CRP composite score into the base model to construct the extended model yielded consistent results.

**Conclusions:**

The SPISE and hs-CRP are not only significantly associated with the risk of collateral circulation impairment, but their inclusion in the base model also leads to a significant improvement in the model’s risk stratification ability.

## 1 Introduction

Chronic total occlusion (CTO) refers to a complete occlusion of a coronary artery with the loss of antegrade blood flow, persisting for at least 3 months (1). Such lesions can lead to persistent myocardial ischemia, significantly increasing the risks of heart failure and mortality. Coronary collaterals serve as conduits that bridge occluded coronary arteries supplied by epicardial or septal arteries, providing an alternative blood supply to the myocardium subtended by an occluded vessel (2). Although the body can form collateral circulation through coronary anastomoses to maintain perfusion in ischemic regions, not all CTO patients can establish sufficient collateral blood flow. Inadequate collateral formation often leads to persistent angina or impaired cardiac function. Therefore, elucidating the metabolic and inflammatory factors that limit collateral growth is of critical importance for optimizing prevention and treatment strategies for CTO patients.

Metabolic abnormalities, particularly insulin resistance, are commonly observed in patients with CTO. Zou et al. studied 338 CTO patients and found that those with poor collateral circulation had significantly higher fasting insulin levels and a higher homeostasis model assessment of insulin resistance (HOMA-IR) (3). Mouquet et al. prospectively enrolled 387 patients with coronary CTO and found that collateral development was significantly impaired in those with metabolic syndrome; in multivariable analysis, elevated fasting glucose and higher HOMA-IR remained independent predictors of poor collateral development (4). This suggests that insulin resistance or hyperinsulinemia may limit collateral vessel formation by impairing endothelial function and inhibiting angiogenesis. However, classic methods for assessing insulin sensitivity require simultaneous measurements of blood glucose and insulin, which limits their widespread application in routine clinical practice. To address this, Paulmichl et al. proposed the single-point insulin sensitivity estimator (SPISE), which demonstrated comparable efficacy to classic methods such as the hyperinsulinemic-euglycemic clamp, quantitative insulin sensitivity check index, and the Matsuda index (5). Since SPISE can be obtained using routine clinical examination and biochemical data, it holds promise as a simple tool for evaluating the relationship between insulin resistance and coronary collateral circulation. Inflammation is another key regulatory factor in collateral vessel formation. As a sensitive marker of the inflammatory response, C-reactive protein (CRP) may impair collateral development by inhibiting endothelial cell function or exacerbating vascular wall inflammation (6). Several studies have shown that inflammation markers such as CRP and the neutrophil/lymphocyte ratio are closely associated with collateral circulation status in CTO patients (7, 8). Mao et al. found that elevated systemic immune-inflammatory index could suppress the differentiation and survival of endothelial progenitor cells, making it an independent predictor of poor collateral circulation in CTO patients with diabetes (9). Additionally, a study involving 220 coronary angiography patients demonstrated that the area under the curve (AUC) for CRP alone in predicting good collateral circulation was 0.777 (10). These findings suggest that inflammation not only promotes the progression of atherosclerosis but may also be involved in collateral vessel remodeling and revascularization failure. To date, no studies have reported on the combined evaluation of SPISE and high-sensitivity C-reactive protein (hs-CRP) in assessing collateral circulation in CTO patients.

This study aims to systematically explore the correlation between SPISE, hs-CRP levels, and CTO collateral circulation, with the goal of providing a simple and reliable reference index for early identification of poor collateral formation risk in clinical practice.

## 2 Methods

### 2.1 Participants and study design

**Figure 1:**
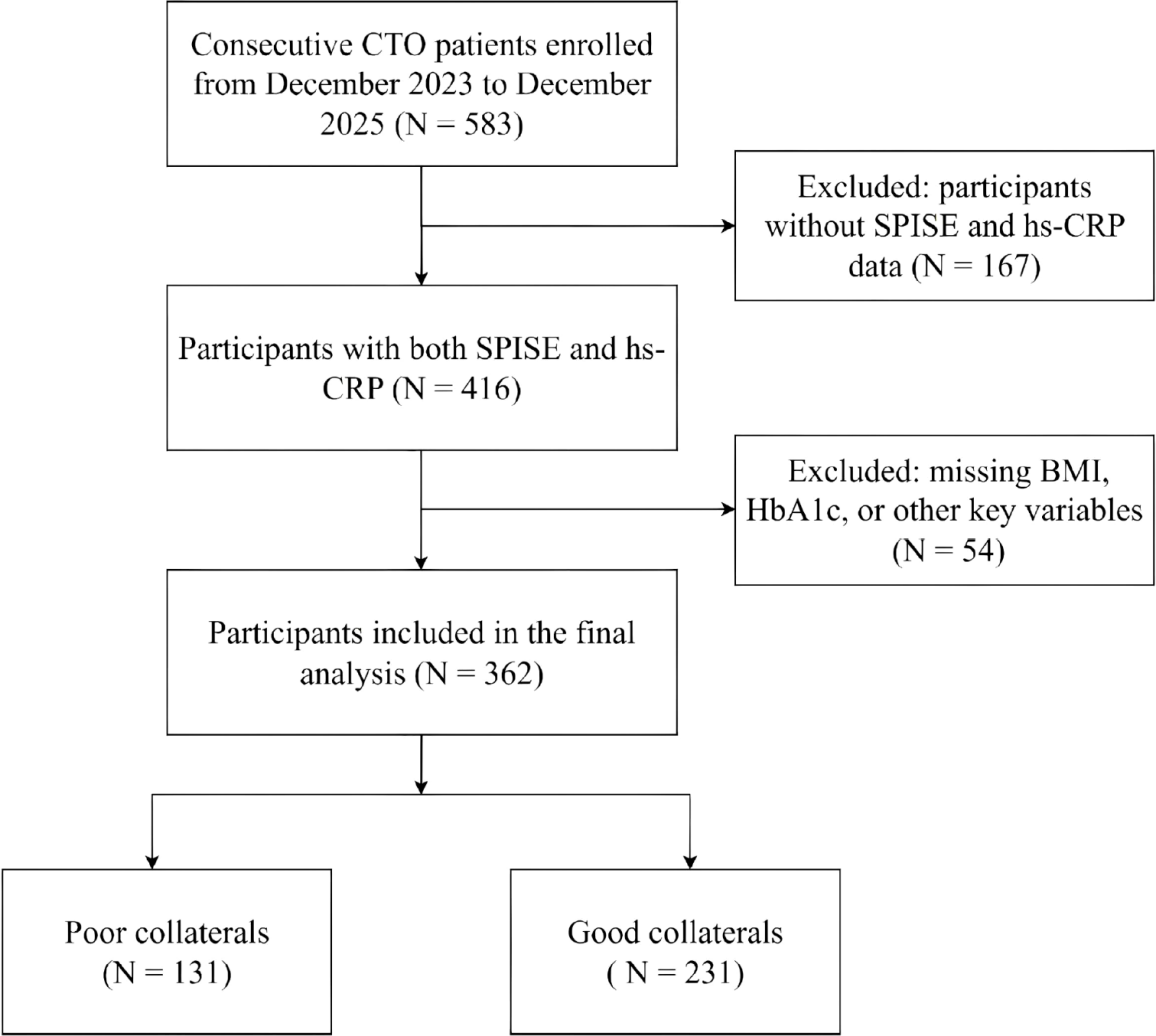
Flowchart of participant selection. CTO, chronic total occlusion; hs-CRP, high-sensitivity C-reactive protein; SPISE, single point insulin sensitivity estimator; BMI, body mass index; HbA1c, glycohemoglobin.

This was a retrospective cross-sectional study conducted at Cangzhou People’s Hospital from December 2023 to December 2025. A total of 583 consecutive patients who underwent first-time coronary angiography and were diagnosed with CTO were initially enrolled. Inclusion criteria were as follows: age ≥18 years; complete occlusion (100% stenosis) of at least one major epicardial coronary artery confirmed by coronary angiography, with occlusion duration ≥3 months determined by prior angiographic records or clinical course, and Thrombolysis in Myocardial Infarction flow grade 0 (1). Of these, 167 patients were excluded due to missing SPISE and hs-CRP data, and an additional 54 patients were excluded due to missing BMI, HbA1c, or other key variables. Ultimately, 362 patients were included in the final analysis. The study protocol was approved by the Ethics Committee of Cangzhou People’s Hospital (K2025-130-02) and was conducted in accordance with the Declaration of Helsinki. Owing to the retrospective design, the requirement for informed consent was waived. The study design is illustrated in **Figure 1**.

### 2.2 Data collection

Demographic, clinical, and laboratory data of patients with CTO were collected from medical records. The collected variables included age, sex, smoking status, drinking status, body mass index (BMI), hypertension, diabetes mellitus, serum uric acid, creatinine, homocysteine, alanine aminotransferase (ALT), glycohemoglobin (HbA1c), total cholesterol (TC), triglycerides (TG), low-density lipoprotein cholesterol (LDL-C), and hs-CRP. BMI was calculated as weight (kg) divided by height (m²). The SPISE score was calculated using the following formula: SPISE = 600 × HDL-C^0.185^/(TG^0.2^ × BMI^1.338^) (5). As the SPISE formula was originally validated using lipid concentrations expressed in mg/dL, fasting HDL-C and TG values were converted from mmol/L to mg/dL (HDL-C: ×38.67; TG: ×88.57) before calculation.

### 2.3 Assessment of collateral circulation impairment

In this study, coronary angiography was used to assess collateral circulation in patients with CTO of the coronary arteries (11, 12). After establishing access using the Seldinger technique and injecting a contrast agent, the Rentrop grading system was applied to qualitatively grade collateral blood flow: Grade 0 indicates no visible collateral blood flow, Grade 1 indicates collateral blood flow reaching only the very proximal or superficial part of the occluded area, Grade 2 indicates collateral blood flow perfusing most of the distal target area of the occlusion, and Grade 3 indicates full collateral perfusion to the main branch of the occlusion, comparable to normal flow. Based on the Rentrop grading results, patients were divided into the poor collateral circulation group (Rentrop grades 0-1) and the good collateral circulation group (Rentrop grades 2-3).

### 2.4 Statistical analysis

Baseline characteristics were presented as mean ± standard deviation or median (interquartile range) for continuous variables, and as counts (percentages) for categorical variables. Between−group comparisons were performed using Student’s t-test, Mann-Whitney U test, or chi-square test, as appropriate. Logistic regression models were used to examine the associations of SPISE and hs-CRP with poor collateral circulation. Model 1 was unadjusted, and Model 2 was adjusted for smoking status, hypertension, diabetes mellitus, homocysteine, TC, LDL-C, and HbA1c. Results were reported as odds ratios (ORs) with 95% confidence intervals (CIs). The variance inflation factor (VIF) was calculated to assess multicollinearity; all VIF values were <5, indicating no significant multicollinearity. Restricted cubic splines (RCS) with knots at the 5th, 35th, 65th, and 95th percentiles were used to explore the dose-response relationships of SPISE and hs-CRP with the risk of poor collateral circulation. Receiver operating characteristic (ROC) curves were generated to evaluate the predictive performance of SPISE and hs-CRP individually, and the AUC was calculated. A base model was constructed including age, sex, smoking status, hypertension, diabetes mellitus, homocysteine, TC, LDL-C, and HbA1c. An extended model was further built by adding SPISE and hs-CRP to the base model. The incremental predictive value was assessed by comparing the AUCs, net reclassification improvement (NRI), and integrated discrimination improvement (IDI) between the two models. A mediation analysis based on bootstrap sampling was performed to evaluate the direct effect of SPISE on poor collateral circulation and the indirect effect mediated by hs-CRP. In sensitivity analyses, weights for SPISE and hs-CRP were determined by proportional scaling according to the absolute regression coefficients from the multivariable logistic model. Using ROC-derived cutoffs, the two indicators were dichotomized and combined into a composite score (range 0-2). This score was incorporated into the base model as a continuous variable, and the NRI and IDI were calculated to assess improvement in model performance. All statistical analyses were performed using R 4.4.2 (R Foundation for Statistical Computing, Vienna, Austria), and P<0.05 (two-tailed) were considered statistically significant.

## 3 Results

### 3.1 Baseline characteristics of the study population

A total of 362 patients with CTO of the coronary arteries were included, with 131 patients (36.2%) assigned to the poor collateral circulation group. No significant differences were observed between the two groups regarding age, sex, drinking status, history of hypertension, serum uric acid, creatinine, and ALT levels (all P > 0.05). Compared with the good collateral circulation group, the poor collateral circulation group had a higher proportion of smokers and patients with diabetes (all P < 0.05). Additionally, the poor collateral circulation group exhibited significantly higher levels of BMI, homocysteine, HbA1c, TC, TG, LDL-C, and hs-CRP, while HDL-C and SPISE were significantly lower (all P < 0.05). Detailed baseline characteristics are shown in **Table 1**.

**Table 1:** Baseline characteristics.

Characteristic	Overall (N = 362)	Good collaterals (N = 231)	Poor collaterals (N = 131)	P
Age, years	66.29 ± 4.18	66.48 ± 4.08	65.96 ± 4.36	0.271
Sex (%)				0.366
Female	155 (42.8%)	103 (44.6%)	52 (39.7%)	
Male	207 (57.2%)	128 (55.4%)	79 (60.3%)	
Smoking status (%)	69 (19.1%)	36 (15.6%)	33 (25.2%)	0.025
Drinking status (%)	83 (22.9%)	58 (25.1%)	25 (19.1%)	0.190
BMI, kg/m^2^	24.67 ± 1.60	24.40 ± 1.59	25.14 ± 1.50	<0.001
Hypertension (%)	218 (60.2%)	141 (61.0%)	77 (58.8%)	0.673
Diabetes mellitus (%)	94 (26.0%)	37 (16.0%)	57 (43.5%)	<0.001
Serum uric acid, μmol/L	308.00 (257.00-364.00)	302.00 (250.00-362.00)	321.00 (265.00-367.00)	0.064
Creatinine, μmol/L	60.30 (53.00-67.10)	60.30 (52.60-66.60)	60.30 (53.00-68.10)	0.537
Homocysteine, μmol/L	16.00 (12.30-24.30)	15.60 (12.00-21.90)	17.70 (13.00-26.90)	0.002
ALT, U/L	19.00 (14.00-29.00)	19.00 (15.00-30.00)	20.00 (14.00-28.00)	0.205
HbA1c, %	5.80 (5.50-6.00)	5.70 (5.50-5.90)	5.90 (5.60-6.10)	<0.001
TC, mmol/L	4.46 ± 1.05	4.35 ± 0.96	4.64 ± 1.17	0.016
TG, mmol/L	1.23 (0.91-1.68)	1.11 (0.84-1.50)	1.41 (1.07-1.97)	<0.001
LDL-C, mmol/L	2.71 ± 0.90	2.60 ± 0.84	2.91 ± 0.97	0.002
HDL-C, mmol/L	1.30 ± 0.33	1.34 ± 0.32	1.21 ± 0.32	<0.001
Hs-CRP, mg/L	1.80 (1.19-2.70)	1.54 (1.12-2.31)	2.65 (1.62-3.56)	<0.001
SPISE	6.66 ± 0.96	6.94 ± 0.94	6.17 ± 0.79	<0.001

hs-CRP, high-sensitivity C-reactive protein; SPISE, single point insulin sensitivity estimator; BMI, body mass index; LAD, left anterior descending artery; LCX, left circumflex artery; RCA, right coronary artery; ALT, alanine aminotransferase; TG, triglycerides; HDL-C, high-density lipoprotein cholesterol; LDL-C, low-density lipoprotein cholesterol; TC, total cholesterol; HbA1c, glycohemoglobin.

### 3.2 Association between SPISE, hs-CRP, and collateral circulation in CTO

In all included participants, SPISE and hs-CRP were significantly associated with collateral circulation status in patients with CTO (**Table 2**). In Model 1, which was unadjusted for confounding factors, SPISE showed a significant negative correlation with poor collateral circulation (OR = 0.354, 95% CI 0.258-0.472, P < 0.001), while hs-CRP was significantly positively correlated with poor collateral circulation (OR = 2.421, 95% CI 1.925-3.098, P < 0.001). In Model 2, which was adjusted for relevant covariates, this association remained significant. Specifically, for each 1-unit increase in SPISE, the risk of poor collateral circulation decreased by 62.9% (OR = 0.371, 95% CI 0.245-0.543, P < 0.001); conversely, for each 1-unit increase in hs-CRP, the risk of poor collateral circulation increased by 113.3% (OR = 2.133, 95% CI 1.617-2.872, P < 0.001).

**Table 2:** ORs (95% CIs) for poor collateral circulation in CTO according to SPISE and hs-CRP.

Variable	Model 1			Model 2		
	OR (95% CI)	P	P for trend	OR (95% CI)	P	P for trend
SPISE	0.354(0.258-0.472)	<0.001	<0.001	0.371(0.245-0.543)	<0.001	<0.001
Hs-CRP	2.421(1.925-3.098)	<0.001	<0.001	2.133(1.617-2.872)	<0.001	<0.001

OR, Odds Ratio; CI, confidence interval; hs-CRP, high-sensitivity C-reactive protein; SPISE, single point insulin sensitivity estimator; LDL-C, low-density lipoprotein cholesterol; TC, total cholesterol; HbA1c, glycohemoglobin.

Model 1 was unadjusted. Model 2 was adjusted for the following covariates: smoking status, hypertension, diabetes mellitus, homocysteine, TC, LDL-C, and HbA1c.

**Figure 2:**
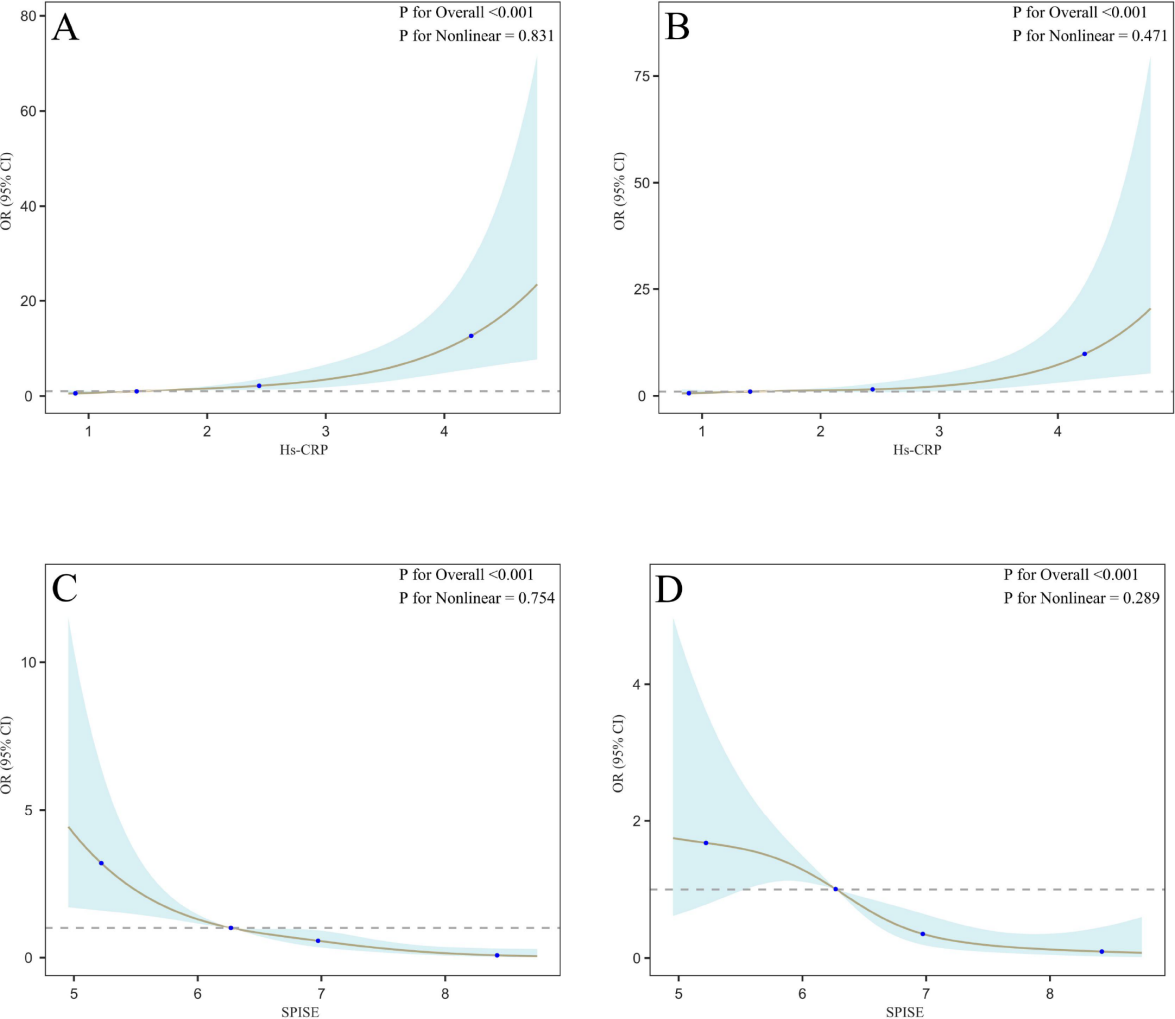
RCS curve for the association between SPISE, hs-CRP, and poor collateral circulation. OR, Odds Ratio; CI, confidence interval; SPISE, single point insulin sensitivity estimator; hs-CRP, high-sensitivity C-reactive protein; LDL-C, low-density lipoprotein cholesterol; TC, total cholesterol; HbA1c, glycohemoglobin. **(A, C)** were unadjusted. **(B, D)** were adjusted for the following covariates: smoking status, hypertension, diabetes mellitus, homocysteine, TC, LDL-C, and HbA1c.

Additionally, trend tests for both indicators indicated a significant dose-response relationship (P for trend < 0.001). **Figure 2** shows the dose-response relationship between SPISE, hs-CRP, and poor collateral circulation in CTO patients, as indicated by the restricted cubic spline curves, which demonstrated a linear relationship across all participants.

To assess whether the predictive value of SPISE and hs-CRP varied according to sex, smoking status, hypertension, and diabetes mellitus, we conducted subgroup analyses (**Table 3**). The associations remained consistent across most subgroups.

**Table 3:** Subgroup analysis.

Variable	N(%)	OR (95% CI)	P	P for interaction
SPISE				0.327
Sex				
Female	155(42.8)	0.256(0.125-0.526)	<0.001	
Male	207(57.2)	0.454(0.272-0.758)	0.003	
Smoking status				0.094
Yes	293(80.9)	0.469(0.299-0.734)	<0.001	
No	69(19.1)	0.075(0.015-0.369)	0.001	
Hypertension				0.264
Yes	144(39.8)	0.313(0.149-0.659)	0.002	
No	218(60.2)	0.389(0.232-0.654)	<0.001	
Diabetes mellitus				0.643
Yes	268(74.0)	0.380(0.227-0.635)	<0.001	
No	94(26.0)	0.465(0.234-0.921)	0.028	
Hs-CRP				
Sex				0.488
Female	155(42.8)	1.989(1.281-3.090)	0.002	
Male	207(57.2)	2.543(1.668-3.877)	<0.001	
Smoking status				0.030
Yes	293(80.9)	2.659(1.875-3.771)	<0.001	
No	69(19.1)	1.204(0.581-2.494)	0.618	
Hypertension				0.057
Yes	144(39.8)	3.398(1.899-6.082)	<0.001	
No	218(60.2)	1.785(1.251-2.547)	0.001	
Diabetes mellitus				0.005
Yes	268(74.0)	2.842(1.923-4.200)	<0.001	
No	94(26.0)	1.205(0.746-1.946)	0.446	

OR, Odds Ratio; CI, confidence interval; SPISE, single point insulin sensitivity estimator; hs-CRP, high-sensitivity C-reactive protein.

### 3.3 Incremental predictive value of SPISE and hs-CRP in participants with collateral circulation impairment

**Figure 3:**
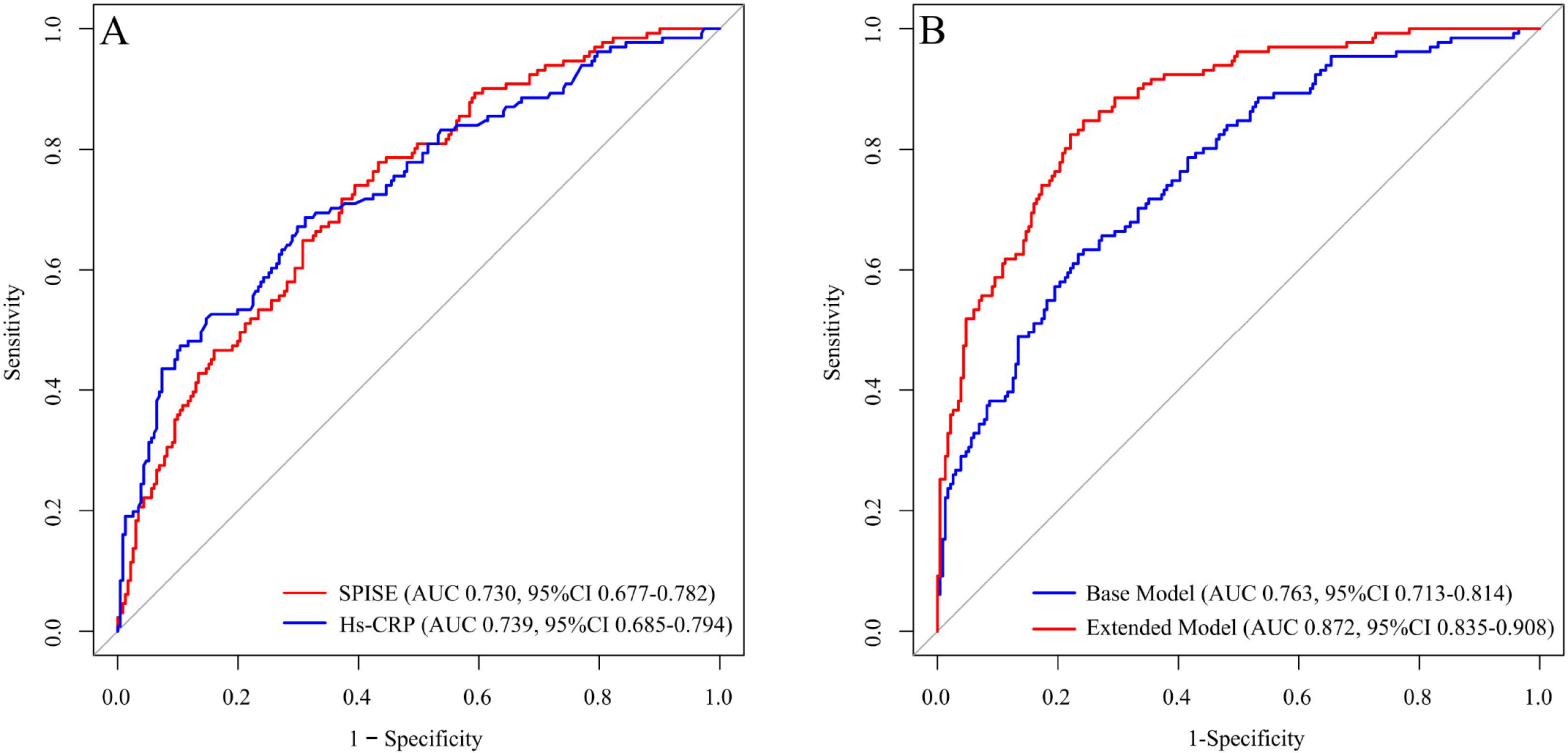
ROC curve comparisons. **(A)** ROC curve comparison between SPISE and hs-CRP. **(B)** ROC curve comparison between the extended model and the base model. AUC, area under the curve; ROC, receiver operating characteristic; CI, confidence interval; hs-CRP, high-sensitivity C-reactive protein; SPISE, single point insulin sensitivity estimator; LDL-C, low-density lipoprotein cholesterol; TC, total cholesterol; HbA1c, glycohemoglobin. The base model included age, sex, smoking status, hypertension, diabetes mellitus, homocysteine, TC, LDL-C, and HbA1c. The SPISE, hs-CRP were added to the base model to construct the extended model.

**Figure 3**, **Table 4** present the ROC curves for SPISE and hs-CRP in predicting collateral circulation impairment in CTO patients. The results show that the AUC for SPISE in predicting collateral circulation impairment was 0.730 (95% CI 0.677-0.782), with an optimal cutoff value of 6.617, sensitivity of 74.0%, and specificity of 60.6%. The AUC for hs-CRP in prediction was 0.739 (95% CI 0.685-0.795), with an optimal cutoff value of 1.915, sensitivity of 68.7%, and specificity of 55.6%.

**Table 4:** ROC analysis of SPISE and hs-CRP for poor collateral circulation.

Variable	AUC (95%CI)	Threshold	Sensitivity	Specificity
SPISE	0.730(0.677-0.782)	6.617	74.0%	60.6%
Hs-CRP	0.739(0.685-0.795)	1.915	68.7%	55.6%

AUC, area under the curve; ROC, receiver operating characteristic; CI, confidence interval; hs-CRP, high-sensitivity C-reactive protein; SPISE, single point insulin sensitivity estimator.

Furthermore, we assessed whether adding SPISE and hs-CRP to the base model, which includes age, sex, smoking status, hypertension, diabetes mellitus, homocysteine, TC, LDL-C, and HbA1c, could enhance the predictive ability for collateral circulation impairment in CTO patients. As shown in **Table 5**, adding SPISE and hs-CRP to the base model significantly improved the predictive capacity of the extended model. The AUC increased from 0.763 (95% CI 0.713-0.814) to 0.872 (95% CI 0.835-0.908), with a DeLong test P < 0.001. The continuous NRI was 0.794 (95% CI 0.592-1.096), and the IDI was 0.197 (95% CI 0.127-0.284), both showing significant increases.

**Table 5:** ROC curve comparison between the extended model based on SPISE, hs-CRP and the base model.

Model	AUC(95%CI)	DeLong test P	NRI (95%CI)	IDI (95%CI)
Base model	0.763(0.713-0.814)	–	–	–
Extended model	0.872(0.835-0.908)	<0.001	0.794(0.592-1.096)	0.197(0.127-0.284)

AUC, area under the curve; ROC, receiver operating characteristic; CI, confidence interval; hs-CRP, high-sensitivity C-reactive protein; SPISE, single point insulin sensitivity estimator; NRI, net reclassification improvement; IDI, integrated discrimination improvement; LDL-C, low-density lipoprotein cholesterol; TC, total cholesterol; HbA1c, glycohemoglobin. The base model included age, sex, smoking status, hypertension, diabetes mellitus, homocysteine, TC, LDL-C, and HbA1c. The SPISE, hs-CRP were added to the base model to construct the extended model.

### 3.4 Mediating role of hs-CRP indicator

Mediation analysis results are presented in **Table 6**. A lower SPISE level was significantly associated with an increased risk of poor collateral circulation in CTO patients. Further analysis revealed that hs-CRP partially mediated this association: in the unadjusted model, the proportion of the association mediated by hs-CRP was 29% (P<0.001); after multivariable adjustment, the mediated proportion remained 27% (P<0.001), suggesting that part of the association between SPISE and poor collateral circulation could be explained by hs-CRP levels.

**Table 6:** Mediation analysis of hs-CRP in SPISE-collateral circulation association.

Effect	Model 1		Model 2	
	Estimate (95% CI)	P	Estimate (95% CI)	P
Hs-CRP				
Total effect	-3.65e-3 (-1.36e-2, -4.88e-4)	<0.001	-2.66e-03 (-1.24e-02, -2.19e-04)	<0.001
Direct effect	-2.60e-3 (-8.40e-3, -3.00e-4)	<0.001	-1.95e-03 (-8.41e-03, -1.61e-04)	<0.001
Indirect effect	-1.10e-3 (-4.80e-3, -1.00e-4)	<0.001	-7.16e-04 (-3.77e-03, -5.31e-05)	<0.001
Indirect/direct effect ratio	4.14e-1 (2.31e-1, 7.41e-1)	<0.001	3.68e-1 (1.95e-1, 6.51e-1)	<0.001
Percentage mediated	0.29 (0.19, 0.43)	<0.001	0.27 (0.16, 0.394)	<0.001

CI, confidence interval; SPISE, single point insulin sensitivity estimator; hs-CRP, high-sensitivity C-reactive protein; LDL-C, low-density lipoprotein cholesterol; TC, total cholesterol; HbA1c, glycohemoglobin. The 95%CIs for the estimates were calculated using the bootstrap method (1000 samples). Model 1 was unadjusted. Model 2 was adjusted for the following covariates: smoking status, hypertension, diabetes mellitus, homocysteine, TC, LDL-C, and HbA1c.

### 3.5 Sensitivity analyses

**Figure 4:**
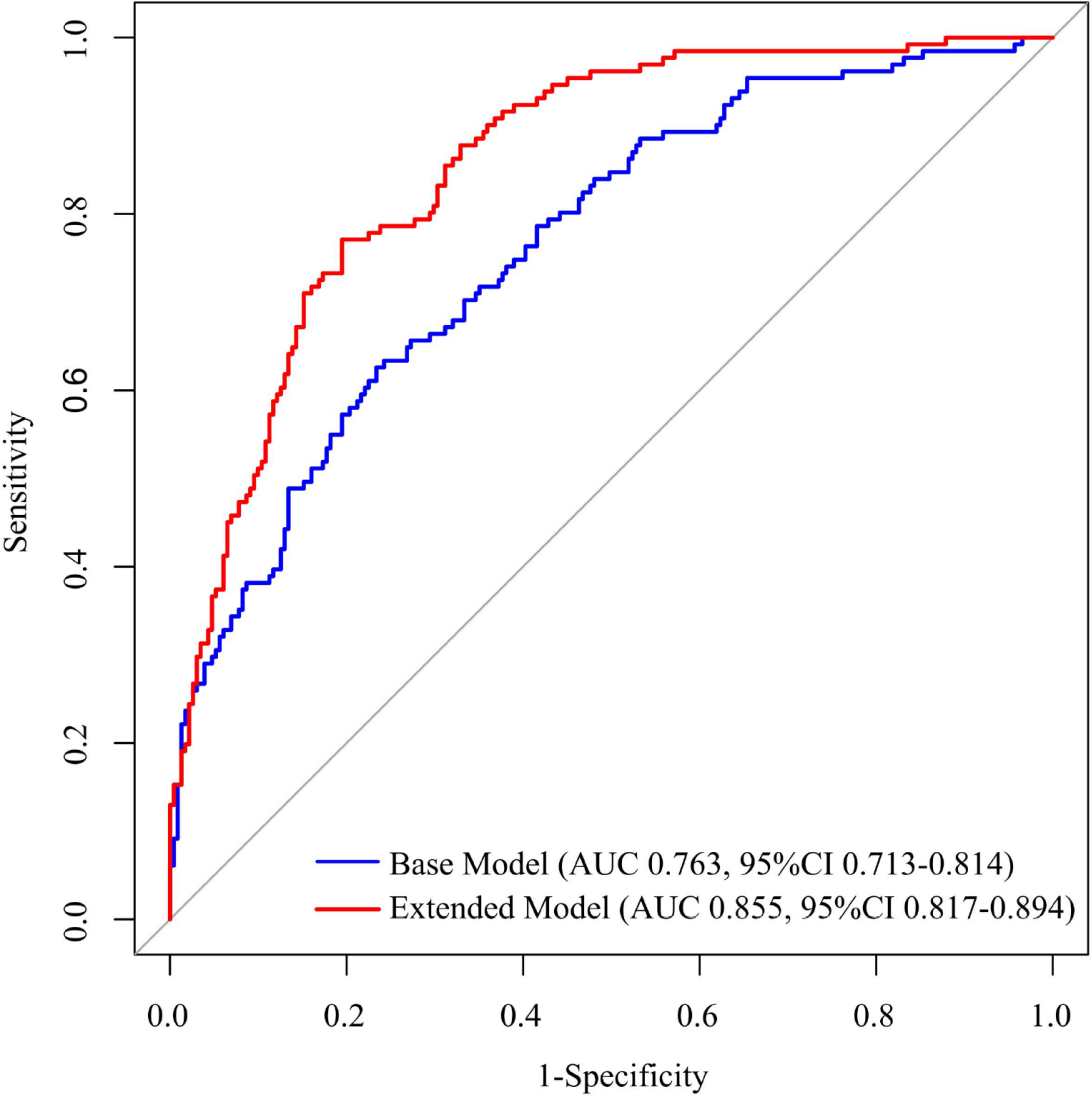
ROC curve comparison between the extended model based on SPISE-hs-CRP Score and the base model. AUC, area under the curve; ROC, receiver operating characteristic; hs-CRP, high-sensitivity C-reactive protein; SPISE, single point insulin sensitivity estimator; LDL-C, low-density lipoprotein cholesterol; TC, total cholesterol; HbA1c, glycohemoglobin. The base model included age, sex, smoking status, hypertension, diabetes mellitus, homocysteine, TC, LDL-C, and HbA1c. The SPISE-hs-CRP composite score was added to the base model to construct the extended model.

Sensitivity analyses were conducted (**Figure 4**). First, based on the absolute values of the regression coefficients for SPISE (β= -0.799) and hs-CRP (β= 0.697) in the multivariable logistic regression model, the indicator weights were determined using a proportional scaling method. The smaller absolute coefficient (|hs-CRP| = 0.697) was assigned 1 point as the reference, and the other indicator (|SPISE| = 0.799) was proportionally calculated (0.799/0.697≈1.146) and rounded to the nearest integer, which also resulted in 1 point. Therefore, both indicators were assigned equal weights (1 point each) in the combined score.

Subsequently, cutoff values derived from the ROC curve were used to classify each indicator: SPISE < 6.617 was defined as high risk, and hs-CRP > 1.915 mg/L was defined as high risk. Each indicator was dichotomized (high risk = 1, low risk = 0), and the sum of the two scores resulted in a new composite score (ranging from 0 to 2 points). This new indicator was incorporated as a continuous variable into the base model, which included age, sex, smoking status, hypertension, diabetes mellitus, homocysteine, TC, LDL-C, and HbA1c, to construct the extended model. A comparison of the two models revealed that the AUC of the extended model was significantly higher than that of the base model (ΔAUC = 0.109, DeLong test P < 0.001). Further analysis showed that the NRI was 0.794 (95% CI 0.592-1.096) and the IDI was 0.197 (95% CI 0.127-0.284), with both 95% CIs not including 0, indicating that the addition of the new indicator significantly improved the model’s reclassification ability and overall predictive performance (**Table 7**).

**Table 7:** ROC curve comparison between the extended model based on SPISE-hs-CRP Score and the base model.

Model	AUC(95%CI)	DeLong test P	NRI (95%CI)	IDI (95%CI)
Base model	0.763(0.713-0.814)	–	–	–
Extended model	0.855(0.817-0.894)	<0.001	0.911(0.695-1.176)	0.156(0.087-0.231)

AUC, area under the curve; ROC, receiver operating characteristic; CI, confidence interval; hs-CRP, high-sensitivity C-reactive protein; SPISE, single point insulin sensitivity estimator; NRI, net reclassification improvement; IDI, integrated discrimination improvement; LDL-C, low-density lipoprotein cholesterol; TC, total cholesterol; HbA1c, glycohemoglobin. The base model included age, sex, smoking status, hypertension, diabetes mellitus, homocysteine, TC, LDL-C, and HbA1c. The SPISE-hs-CRP score was added to the base model to construct the extended model.

## 4 Discussion

In this study, we explored the role of SPISE and hs-CRP in collateral circulation formation in CTO patients. The main findings include: After adjusting for traditional risk factors, both decreased SPISE and elevated hs-CRP were identified as independent predictors of poor collateral circulation; both indicators were linearly related to collateral circulation; and their combination improved the predictive ability for poor collateral circulation. In addition, we undertook an exploratory mediation analysis to examine whether hs-CRP mediates the association between SPISE and collateral circulation. Although the analysis suggested that hs-CRP may act as a statistical intermediary, the cross-sectional nature of our data precludes establishing temporal order, so these mediation findings should be interpreted as exploratory rather than causal. Overall, our results suggest that insulin resistance and inflammation may act synergistically in coronary collateral remodeling.

Collateral circulation refers to the vascular structures formed within the coronary arteries or between different coronary arteries, aimed at maintaining blood supply and vitality to the myocardial tissue distal to the lesion in the event of significant stenosis or complete occlusion leading to circulatory disruption (13). Good coronary collateral circulation plays a key role in preventing ischemic events. We observed that decreased SPISE was associated with insufficient collateral circulation in CTO patients, which is consistent with previous studies using traditional insulin resistance indicators. A study by Xiao et al., involving 2,691 patients, showed that an elevated triglyceride-glucose (TyG) index was associated with CTO risk and may serve as a meaningful predictor for CTO (14). Gürses et al. were the first to demonstrate that a high TyG index is an independent risk factor for an elevated J-CTO score in CTO patients (15). Zhu et al. noted that for every unit increase in TyG index, the risk of poor collateral circulation increased 5.104 times, with an AUC of 0.779 for predicting poor collateral circulation (16). Additionally, a study of 331 patients with at least one CTO lesion who underwent percutaneous coronary intervention (PCI) found that TyG index independently predicted major adverse cardiovascular and cerebrovascular events after CTO-PCI (17). Insulin resistance may suppress collateral circulation development through multiple mechanisms. Firstly, the high insulin levels associated with insulin resistance can promote atherosclerosis and increase oxidative stress, reducing endothelial nitric oxide synthase activity, inhibiting endothelium-dependent vasodilation, and decreasing angiogenesis potential (18). Secondly, insulin resistance can reduce the number and activity of endothelial progenitor cells, impairing their repair and regenerative functions. Insulin resistance is inversely related to the number of colony-forming units of endothelial cells, resulting in reduced collateral density during myocardial ischemia (4). The correlation between TyG index and arterial stiffness markers also reflects the detrimental effects of insulin resistance on vascular health. Since SPISE is highly correlated with insulin resistance, a low SPISE value can be considered a comprehensive manifestation of hyperinsulinemia and metabolic disorders, which may explain its association with poor collateral circulation. The SPISE is derived from fasting lipid and body mass index values, does not require insulin measurement, and therefore offers a simple surrogate for insulin sensitivity. A pilot study involving patients with metabolic syndrome reported that SPISE showed a higher ROC-AUC and better diagnostic performance than HOMA-IR, the TG/HDL-C ratio, and hs-CRP for identifying insulin resistance (19). Thus, the association we observed between decreased SPISE and poor collateral circulation underscores the potential clinical value of SPISE in cardiovascular research. Our findings also extend previous TyG-oriented work by re-centering the discussion on SPISE and demonstrating that the combination of SPISE and hs-CRP improves prediction of poor collateral formation.

Inflammation plays a central role in the pathogenesis of atherosclerosis and coronary collateral vessel formation. Atherosclerosis is essentially a chronic inflammatory process, characterized by inflammatory cell infiltration, cytokine release, and endothelial dysfunction. These changes not only promote plaque formation and progression but may also hinder the compensatory expansion and maturation of collateral vessels. In 2013, Ayhan et al. were the first to use inflammation markers in assessing coronary collateral circulation, finding that the neutrophil/lymphocyte ratio and mean platelet volume were associated with poor collateral circulation (20). Subsequently, several studies further confirmed the association between inflammation markers and collateral circulation: a study involving 354 patients with stable coronary artery disease examined the relationship between the CRP to albumin ratio and coronary collateral circulation (21). Moreover, the systemic immune-inflammatory index has also been reported as an independent predictor for collateral circulation development (22). In a retrospective study of 320 patients, Aktaş et al. found that the neutrophil percentage and the albumin ratio were independently associated with collateral circulation in CTO patients with acute myocardial infarction (23). Inflammation plays a central role in the pathophysiology of collateral circulation (24). Research has shown that increased nitric oxide production promotes endothelial cell proliferation and migration, while CRP-mediated inflammation inhibits nitric oxide production, thereby hindering angiogenesis (25, 26). Zorkun et al. confirmed that higher levels of hs-CRP are a determinant of collateral circulation in coronary artery disease patients (27). Furthermore, CRP can inhibit vascular endothelial growth factor (VEGF)-mediated endothelial cell migration, with VEGF being a key factor in regulating endothelial cell proliferation (28). Our findings further support an independent association between hs-CRP and collateral circulation in patients with CTO, suggesting that inflammation may negatively influence collateral vessel formation. Furthermore, the association between hs-CRP and poor collateral circulation was significant in diabetic patients but not in non-diabetic patients, suggesting that diabetes may amplify the detrimental effect of inflammation on collateral formation. In the diabetic state, chronic hyperglycemia can induce a pro-inflammatory endothelial phenotype, enhance oxidative stress, and reduce nitric oxide bioavailability, thereby rendering the coronary microvasculature more susceptible to the anti-angiogenic actions of CRP. Conversely, the lower baseline inflammatory burden in non−diabetic patients may attenuate this association. More importantly, the combined assessment of SPISE and hs-CRP significantly improved the prediction of poor collateral circulation, indicating that metabolic dysfunction and systemic inflammation may provide complementary information in risk stratification. This interpretation is consistent with evidence from a large Korean population-based cross-sectional study, in which individuals with both elevated hs-CRP and overweight/obesity had nearly three-fold higher odds of insulin resistance than those without these exposures (29). Although this evidence was not derived from a CTO population, it supports the broader concept that inflammatory and metabolic disturbances may interact biologically, providing a plausible context for the improved predictive value of the SPISE–hs-CRP combination observed in our study.

## 5 Limitations

However, there are several limitations in this study. First, this study is a single-center, retrospective cross-sectional design with a relatively limited sample size, which may introduce selection bias and thus limit the generalizability of the results to a broader population. Second, as a retrospective study, it is inherently susceptible to information bias, which may compromise a comprehensive assessment of the study outcomes. Third, although we adjusted for multiple potential confounders in the multivariable logistic regression model, residual confounding inherent in retrospective studies cannot be fully excluded. In particular, medications such as statins, antidiabetic agents (e.g., metformin, GLP-1 receptor agonists), and RAAS blockers are known to modulate both hs-CRP levels and insulin sensitivity. However, detailed data on the use, dosage, and duration of these medications were not consistently documented in the medical records and therefore could not be incorporated into the adjusted models. Fourth, no *a priori* sample size calculation was performed; the sample was determined by the number of eligible patients within the study period, and statistical power may be limited for certain subgroup analyses. Fifth, the mediation analysis, while statistically significant, is based on cross-sectional data, which precludes the determination of a temporal sequence between SPISE changes, hs-CRP elevation, and the development of impaired collateral circulation. Therefore, these findings should be considered exploratory and hypothesis-generating for future prospective studies, rather than as confirmatory of a causal pathway. Future large-scale, multi-center, prospective cohort studies are needed to further validate and refine the conclusions of this study.

## 6 Conclusion

In this study, lower SPISE and higher hs-CRP were independently associated with impaired collateral circulation, and their combined assessment significantly improved risk stratification. An exploratory mediation analysis suggested that hs-CRP may partially link SPISE to collateral impairment, but causality cannot be inferred. These findings support the potential value of integrating SPISE and hs-CRP into the clinical evaluation of CTO patients, and prospective studies are warranted to validate and extend these observations.

## Data Availability

The original contributions presented in the study are included in the article/Supplementary Material. Further inquiries can be directed to the corresponding author.
